# Association between fibrinogen–albumin ratio and outcomes in myocardial infarction: A systematic review and meta-analysis

**DOI:** 10.12669/pjms.42.4.14991

**Published:** 2026-04

**Authors:** Peng Xu, Hua Xu, Si Shen, Jiahui Zhu, Wenxi Shen

**Affiliations:** 1 Peng Xu Department of Emergency, Huzhou Central Hospital, Huzhou, Zhejiang Province 313000, P.R. China; 2 Hua xu Department of Emergency, Huzhou Central Hospital, Huzhou, Zhejiang Province 313000, P.R. China; 3 Si Shen Department of ICU, Huzhou Central Hospital, Huzhou, Zhejiang Province 313000, P.R. China; 4 Jiahui Zhu Department of Emergency, Huzhou Central Hospital, Huzhou, Zhejiang Province 313000, P.R. China; 5 Wenxi Shen Department of Emergency, Huzhou Central Hospital, Huzhou, Zhejiang Province 313000, P.R. China

**Keywords:** Albumin, Biomarker, Fibrinogen, Heart attack, Inflammation

## Abstract

**Background & Objective:**

The fibrinogen–albumin ratio (FAR) is an emerging biomarker that reflects systemic inflammation and coagulation balance. Its prognostic significance in myocardial infarction (MI) remains uncertain. This systematic review and meta-analysis evaluated the association between FAR and clinical outcomes in MI patients.

**Methodology:**

A comprehensive search of PubMed, Embase, Scopus, and Web of Science was conducted up to 10^th^ November 2025 for studies reporting the relationship between FAR and outcomes in MI. Pooled odds ratios (OR) with 95% confidence intervals (CI) were calculated using random-effects models.

**Results:**

Six studies involving 3,007 MI patients were included. Five studies examined ST-elevation MI and one assessed MI with non-obstructive coronary arteries. Pooled analysis revealed that elevated FAR significantly predicted major adverse cardiac events (MACE) (OR: 2.62; 95% CI: 1.88–3.65; I²=4%) and no-reflow phenomenon (OR: 2.01; 95% CI:1.75–2.30; I²=88%). However, FAR was not significantly associated with mortality (OR: 1.03; 95% CI: 0.99–1.06; I²=90%).

**Conclusions:**

Elevated FAR is linked with increased risks of MACE and no-reflow after reperfusion in MI patients, but not with mortality. FAR may be a simple, affordable marker for early prognostic evaluation, requiring further validation in large prospective studies.

**Registration No.:** PROSPERO (CRD420251229169).

## INTRODUCTION

Myocardial infarction (MI) remains a principal cause of mortality globally, notwithstanding significant advancements in diagnostic and interventional methodologies.^1^,^2^ Among its various clinical presentations, ST-segment elevation MI (STEMI) is associated with the highest rates of mortality and unfavourable outcomes.^2^ Although primary percutaneous coronary intervention (PCI) has substantially enhanced survival rates, a considerable proportion of patients continue to encounter recurrent ischemic events, heart failure, and mortality during follow-up periods.^3^ Consequently, there is an ongoing research to identify reliable and cost-effective biomarkers that can facilitate early risk stratification and prognostic evaluation.^4^

Inflammation and thrombosis are key aspects of the pathophysiology of MI, influencing plaque rupture, microvascular blockage, and heart tissue damage.^5^ The no-reflow phenomenon, which involves inadequate blood flow despite successful revascularisation, is a common complication that worsens patient outcomes. Increased inflammatory response and blood flow abnormalities are known to contribute to this condition.^6^ Blood-based inflammatory markers, such as the neutrophil-lymphocyte ratio, platelet-lymphocyte ratio, prognostic nutritional index, and geriatric nutritional risk index, have demonstrated some predictive value; however, their practical use in clinical settings remains limited.^4^,^7^ Evidence on the validity of such biomarkers is especially important for nursing personnel as they are simple and can be readily used by them to prognosticate patients.

Fibrinogen and albumin are recognised as important markers of inflammation and nutritional status, reflecting systemic inflammation and blood viscosity.^8^ Elevated fibrinogen levels encourage platelet clumping and clot formation, whereas low albumin levels suggest poor nutrition and endothelial dysfunction.^9^ The fibrinogen-to-albumin ratio (FAR), combining these two factors, has recently been identified as a new inflammatory marker with possible implications for cardiovascular health.^10^ Several studies have investigated its prognostic value in MI patients, but their findings have been inconsistent.^11^,^12^ Consequently, this systematic review and meta-analysis aim to thoroughly assess the prognostic significance of the FAR in MI patients, particularly regarding its relationship with mortality, major adverse cardiac events (MACE), and the no-reflow phenomenon.

## METHODOLOGY

Two reviewers (PX, HX) independently conducted a thorough search across PubMed, Embase, Scopus, and Web of Science to find relevant studies. They also searched Google Scholar and preprint servers for gray literature. The search included all publications from January 1, 1990, to November 10, 2025, with no language restrictions. Any disagreements between reviewers were resolved by a third author (SS). The search combined controlled vocabulary (MeSH/Emtree) terms and free-text keywords using Boolean operators “AND” and “OR.” Detailed search strategies for each database are available in Supplementary Table-I.

**Supplementary Table-I:** Search strategy.

Database	Search strategy
PubMed	(“Myocardial Infarction”[Mesh] OR “myocardial infarction”[Title/Abstract] OR “acute myocardial infarction”[Title/Abstract] OR AMI[Title/Abstract] OR STEMI[Title/Abstract] OR NSTEMI[Title/Abstract] OR “heart attack”[Title/Abstract]) AND (“fibrinogen-albumin ratio”[Title/Abstract] OR “fibrinogen to albumin ratio”[Title/Abstract] OR “fibrinogen/albumin ratio”[Title/Abstract] OR “fibrinogen albumin ratio”[Title/Abstract] OR ((fibrinogen[Title/Abstract] AND albumin[Title/Abstract] AND ratio[Title/Abstract])) OR FAR[Title/Abstract]))
Embase	(’myocardial infarction’/exp OR ’myocardial infarction’:ti,ab OR ’acute myocardial infarction’:ti,ab OR AMI:ti,ab OR STEMI:ti,ab OR NSTEMI:ti,ab OR ’heart attack’:ti,ab) AND ((fibrinogen:ti,ab NEAR/3 albumin:ti,ab NEAR/3 ratio:ti,ab) OR ’fibrinogen-albumin ratio’:ti,ab OR ’fibrinogen/albumin ratio’:ti,ab OR ’fibrinogen to albumin ratio’:ti,ab OR FAR:ti,ab)
Scopus	TITLE-ABS-KEY((“myocardial infarction” OR “acute myocardial infarction” OR AMI OR STEMI OR NSTEMI OR “heart attack”) AND ((“fibrinogen” W/3 “albumin” W/3 “ratio”) OR “fibrinogen-albumin ratio” OR “fibrinogen/albumin ratio” OR “fibrinogen to albumin ratio” OR FAR))
Web of Science	TS=((“myocardial infarction” OR “acute myocardial infarction” OR AMI OR STEMI OR NSTEMI OR “heart attack”) AND ((“fibrinogen” NEAR/3 “albumin” NEAR/3 “ratio”) OR “fibrinogen-albumin ratio” OR “fibrinogen/albumin ratio” OR “fibrinogen to albumin ratio” OR FAR))

After completing the search, all records were deduplicated and independently screened by two reviewers (PX, HX) through a two-step process: initial title/abstract screening, then full-text review. A third reviewer resolved any conflicts (SS). Additionally, reference lists of included studies were hand-searched to find more eligible articles.

The current review protocol was registered prospectively on PROSPERO (CRD420251229169). The review was designed and reported in accordance with the PRISMA 2020 guidelines.^13^

### Eligibility Criteria:

Studies were included based on the following PECO framework:

- Population: Patients diagnosed with MI (STEMI or NSTEMI).
- Exposure: Elevated FAR.
- Comparison: Lower FAR.
- Outcomes: Mortality, MACE, and no-reflow phenomenon.

### Inclusion Criteria:

- It included observational study designs that reported adjusted or unadjusted estimates of the association between FAR and clinical outcomes.

### Exclusion criteria:

- Non-original articles (reviews, editorials, or case reports).
- Studies not exclusively on MI patients.
- Duplicate datasets. In such a case, only the study with the largest sample was retained.

### Data Extraction and quality assessment:

Data extraction was performed in duplicate using a predesigned form. Extracted details included first author, year of publication, country, study design, sample size, patient demographics (age, sex), type of MI, FAR measurement and cut-off, follow-up duration, and outcomes of interest (mortality, MACE, and no-reflow phenomenon).

The quality of the included cohort studies was evaluated using the Newcastle–Ottawa Scale (NOS).^14^ This scale assesses three areas: selection, comparability, and outcome measurement, and a study can score up to nine points. Studies with a score of seven or higher were classified as high quality. Any disagreements in scoring were resolved through consensus.

### Statistical Analysis:

A meta-analysis was performed using Comprehensive Meta-analysis software (Version 3). Pooled effect estimates were combined in a random-effects model and expressed as odds ratios (OR) with 95% confidence intervals (CI). Heterogeneity was quantified using the I² statistic, with values of <50% indicating low heterogeneity and >50% signifying substantial heterogeneity. Sensitivity analysis was conducted by sequential exclusion of each study to assess result stability. Due to limited data, publication bias could not be assessed. GRADE was used to assess the certainty of evidence.

When both unadjusted and adjusted results were accessible, the adjusted effect estimates were preferred. Among the reviewed studies, adjusted ORs were used in two out of four mortality analyses, both MACE analyses, and one out of three no-reflow analyses; the other estimates came from unadjusted data in the original reports. Since no study reported hazard ratios (HR), there was no need for HR-to-OR conversion. All combined effect measures were odds ratios (OR), eliminating the need for data harmonization. Where OR with 95% CI were unavailable, they were calculated from raw data.

## RESULTS

**Fig.1:**
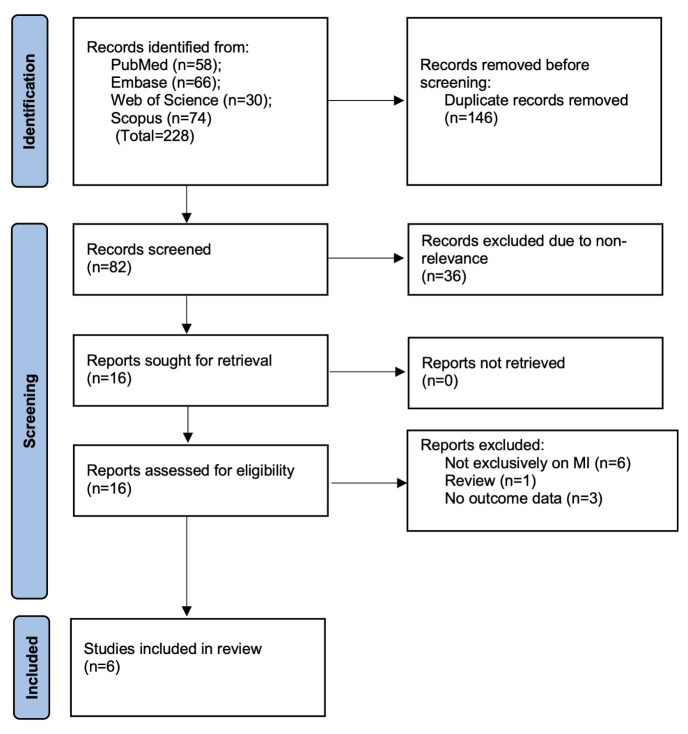
Study flowchart.

The PRISMA flowchart illustrating the study selection process is shown in Fig.1. The initial database search retrieved a total of 228 records. After removing 146 duplicates, 82 unique articles remained and were screened based on titles and abstracts. Of these, 36 were excluded due to irrelevance. The remaining 16 articles underwent full-text review. During this review, six studies were excluded: one was a review article, three lacked relevant outcome data, and two did not focus solely on MI populations. Six studies were included.^6^,^9^,^11^,^12^,^15^,^16^

Across six studies, a total of 3,007 patients with MI were involved (Table-I), with four studies conducted in China, one in Turkey, and one in Egypt. Among these, four studies were retrospective, and two were prospective cohorts. Participants’ ages, reported as either the mean or median, ranged from 57 to 64 years, with males comprising the majority. The prevalence of diabetes mellitus varied widely from 15.6% to 82.1%, while hypertension rates ranged from 26.7% to 55%. Regarding MI subtypes, five studies involved patients with STEMI, and one focused on MINOCA (MI with non-obstructive coronary arteries). All patients underwent PCI. The prognostic stratification used FAR cut-off values that differed among studies, ranging from 0.076 to 0.082; however, three studies did not specify a threshold. When available, median follow-up periods extended up to 60 months. NOS score of the studies varied from 8-9 indicating high quality data.

**Table-I:** Details of included studies.

Study ID	Country	Design	Type of MI	Sample size	Age (Years)	Males (%)	DM (%)	HT (%)	FAR cut-off	Follow-up (months)	NOS score
Fang 2025	China	R	MINOCA	1031	57	76.9	21.3	53.4	0.082	42.3	S-4 C-2 O-3
Kaplangoray 2023	Turkey	P	STEMI	167	59.4	57.5	40.1	33.3	0.076	NR	S-4 C-2 O-2
Refaat 2021	Egypt	R	STEMI	400	59	71	56	55	NR	NR	S-4 C-2 O-2
Liu 2021	China	P	STEMI	424	63	61.6	15.6	26.7	NR	60	S-4 C-2 O-3
Zhao 2019	China	R	STEMI	510	61.1	78.9	29.4	48.4	NR	1	S-4 C-2 O-2
Xiao 2019	China	R	STEMI	475	64	76.4	82.1	51.4	0.08	14.4	S-4 C-2 O-3

MI, myocardial infarction; MINOCA, MI with non-obstructive coronary arteries; STEMI, ST elevated MI; New Castle Ottawa Scale; FAR, fibrinogen-albumin ratio; DM, diabetes mellitus; HT, hypertension; R, retrospective; S, selection of studies; C, comparability; O, outcome assessment; NR, not reported; Prospective.

**Fig.2:**
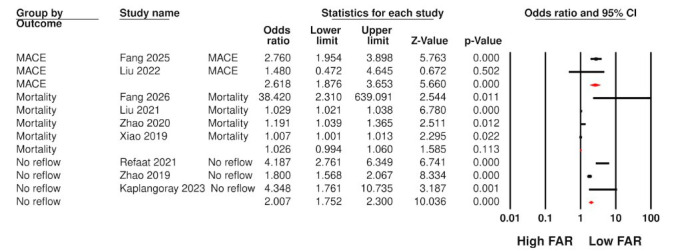
Meta-analysis of mortality, MACE, and no-reflow between high and low FAR patients with MI.

Meta-analysis results are shown in Fig.2. Four studies reported data on mortality. Pooled analysis showed that high FAR did not predict mortality in patients with MI (OR: 1.026 95% CI: 0.994, 1.060 I^2^=90%). On sensitivity analysis, the results remained non-significant on exclusion of individual studies. Data on MACE was reported by two studies. Meta-analysis of two studies showed that high FAR was significantly predictive of MACE (OR: 2.618 95% CI: 1.876, 3.653 I^2^=4%). Three studies reported data on no-reflow phenomenon. Pooled analysis indicated that high FAR was associated with significantly high risk of no-reflow after PCI in patients with MI (OR: 2.007 95% CI: 1.752, 2.30 I^2^=88%). Sensitivity analysis failed to change the significance of results on exclusion of individual studies. The certainty of evidence was “very low” on GRADE.

## DISCUSSION

In summary, this meta-analysis of six studies focusing solely on MI patients indicates that FAR may predict MACE and the no-reflow phenomenon. However, the current pooled evidence does not demonstrate a statistically significant association between FAR and mortality (OR: 1.026; 95% CI: 0.994–1.060). Importantly, this estimate is close to statistical significance and is accompanied by substantial heterogeneity (I²=90%), based on only four studies. Therefore, definitive conclusions regarding the relationship between FAR and mortality cannot be drawn at present.

The current results are consistent with other studies examining the prognostic value of FAR in cardiovascular diseases. A previous meta-analysis by Desai et al.^17^ has shown that high FAR is associated with increased risks of all-cause mortality, cardiac mortality, MACE, and no-reflow in coronary artery disease patients undergoing PCI. While their study included all types of coronary artery disease patients, our review focused solely on MI to ensure homogeneity of the patient population and generate more targeted results. Guo et al.^18^ in a larger study of 1880 patients with heart failure, demonstrated that increasing FAR correlates with higher risks of 28-day and 3-month mortality. Han et al.^19^ observed a significant positive association between high FAR and all-cause mortality in patients with atrial fibrillation. Similarly, Sun et al.^20^ found that high pre-treatment FAR in acute ischaemic stroke patients undergoing thrombolysis relates to early neurological deterioration. A propensity score-matched study by Liu et al.^21^ revealed that high FAR is linked to haemorrhagic transformation in ischaemic stroke patients receiving thrombolytic therapy.

The predictive capacity of FAR in MI extends beyond MACE, mortality, and no-reflow. In fact, studies have shown that FAR may also predict other outcomes. Bao et al.,^22^ in their study involving 670 patients with MI, noted that FAR is a reliable tool for predicting new-onset atrial fibrillation in such patients. Another study has demonstrated that MI patients with high FAR face a 1.5 times greater risk of acute kidney injury.^23^

Interestingly, FAR is not only predictive of outcomes in cardiovascular diseases but is also associated with disease severity. A study of 2825 patients undergoing coronary angiography showed that FAR was directly linked with the severity of coronary artery disease.^10^ Wang et al.^24^ have demonstrated that FAR is independently and significantly associated with left ventricular systolic dysfunction in patients with acute coronary syndrome. Another study indicated that FAR predicts cerebral small vessel disease burden in patients with transient ischaemic attack.

The notable heterogeneity seen in the mortality (I²=90%) and no-reflow (I²=88%) analyses warrants careful examination. Several factors might account for this variability. Firstly, differences in study design—such as retrospective versus prospective cohorts—and sample sizes could have affected effect estimates. Secondly, Furthermore, one included study enrolled patients with MINOCA, a condition with distinct mechanisms such as coronary vasospasm, microvascular dysfunction, or plaque erosion rather than classic plaque rupture seen in STEMI. Combining MINOCA and STEMI populations may reduce biological homogeneity and contribute to heterogeneity in pooled estimates. Thirdly, FAR cut-off values differed across studies; three did not specify a clear threshold, potentially causing inconsistencies in risk assessment. Fourth, follow-up periods varied significantly, especially for mortality, from short-term in-hospital to long-term follow-ups up to 60 months. Additionally, differences in adjusting for confounders and in the definitions and angiographic evaluation of no-reflow likely contributed to the heterogeneity.

The FAR has prognostic importance in MI because it reflects two crucial biological processes: inflammation and coagulation.^19^ Fibrinogen levels rise during acute inflammation and are essential for platelet aggregation and clot formation, which can lead to thrombosis and blood vessel blockages. Elevated fibrinogen also raises blood viscosity, causes oxidative stress, and promotes smooth muscle cell growth, all of which accelerate and destabilise atherosclerotic plaques.^25^ Conversely, low serum albumin, a negative acute-phase protein, indicates systemic inflammation, endothelial damage, and reduced antioxidant defences. Albumin normally helps control vascular permeability, maintain blood oncotic pressure, and inhibit platelet activation by modulating nitric oxide and prostacyclin levels. When albumin decreases, it can result in increased vascular permeability, oxidative stress, and endothelial injury, raising the risk of plaque rupture and microvascular damage.^18^ A high FAR, indicating increased fibrinogen and lower albumin, suggests a heightened inflammatory and hypercoagulable state, which heightens the likelihood of adverse cardiovascular events.^22^

Clinically, this proinflammatory and prothrombotic environment directly influences outcomes like mortality, MACE, and the no-reflow phenomenon. In acute MI, elevated FAR levels contribute to microvascular obstruction by causing endothelial injury, fibrin accumulation, and platelet clumping, which hinder myocardial reperfusion even after revascularisation.^6^ This microvascular damage is a key factor in the no-reflow phenomenon, strongly linked to larger infarct sizes, adverse remodelling, and poorer short-term outcomes.^16^ Systemic inflammation and hemodynamic stress further worsen ventricular dysfunction and heart failure, raising the risk of mortality and MACE during follow-up. Higher FAR levels are also associated with increased SYNTAX scores, indicating more severe and complex coronary lesions.^10^ Thus, FAR serves as a marker of inflammation, coagulation activity, vascular health, microcirculatory function, and long-term prognosis in MI.

### Limitations:

It includes a small number of studies, mostly single-centre cohorts, with potential residual confounding. Heterogeneity in FAR measurement methods, thresholds, and timing across studies may have affected effect estimates. Variability in statistical adjustments and possible publication bias, especially for null results, also exists. Most studies involved Asian populations, limiting generalizability. Differences in outcome definitions and the focus on baseline FAR, without assessing changes over time, add to variability. Due to the limited data available, detailed subgroup analyses and meta-regression could not be conducted. An additional limitation relates to the pooling of adjusted and unadjusted effect estimates. Although the analysis prioritized adjusted estimates, not all studies furnished multivariable-adjusted results. Combining both adjusted and unadjusted ORs could lead to bias because unadjusted estimates do not account for confounders like age, comorbidities, infarct severity, and treatment variations. The limited number of studies per outcome prevented subgroup analyses based solely on adjusted estimates. Consequently, the pooled results should be interpreted cautiously, and future research should aim to report standardized multivariable-adjusted associations.

This study is, to our knowledge, the first systematic review and meta-analysis to specifically assess the prognostic significance of FAR in patients with myocardial infarction, thereby improving clinical specificity. Conducted following PRISMA 2020 guidelines and with prospective PROSPERO registration, it included several relevant outcomes such as mortality, MACE, and no-reflow. The review preferentially used adjusted estimates when available and performed sensitivity analyses to verify robustness. However, larger, multicentre prospective studies with standardized FAR measurement protocols and consistent cut-off values are necessary. Future research should analyse FAR in different MI subtypes, explore serial FAR measurements, and evaluate whether FAR adds predictive value beyond existing risk scores before recommending routine clinical use.

This review suggests that the FAR can serve as a simple and inexpensive biomarker for early risk assessment in patients with MI. Elevated FAR can be used by clinicians and nursing personnel to identify high-risk patients for MACE and no-reflow after reperfusion, guiding closer monitoring and aggressive prevention. Incorporating FAR into prognostic models could improve decision-making and personalised treatment, especially in resource-limited settings.

## CONCLUSIONS

This systematic review and meta-analysis demonstrate that an elevated FAR is significantly associated with adverse outcomes in patients with MI, including increased incidence of MACE and greater risk of no-reflow phenomenon following reperfusion therapy. However, current evidence does not demonstrate a statistically significant association with mortality. Given the high heterogeneity and limited number of available studies, this finding should be interpreted cautiously, and further robust investigations are warranted.
